# Susceptibility of Some *Corylus avellana* L. Cultivars to *Xanthomonas arboricola* pv. *corylina*

**DOI:** 10.3389/fpls.2021.800339

**Published:** 2021-12-17

**Authors:** John Bryan Webber, Sugae Wada, Virginia O. Stockwell, Nik G. Wiman

**Affiliations:** ^1^Department of Horticulture, Oregon State University, Corvallis, OR, United States; ^2^Horticultural Crops Research Unit, United States Department of Agriculture, Agricultural Research Service, Corvallis, OR, United States; ^3^Oregon State University, North Willamette Research and Extension Center, Aurora, OR, United States

**Keywords:** hazelnut, disease screening, inoculation, tissue culture, *in vitro*, *in vivo*, eastern filbert blight, bacterial blight

## Abstract

Bacterial blight of hazelnut (*Corylus avellana* L.) is caused by *Xanthomonas arboricola* pv. *corylina* (Xac). In the past, bacterial blight has been a key disease impacting the Oregon hazelnut industry where 99% of the United States hazelnut crop is grown. The disease is re-emerging in young orchards, as acreage of newly released hazelnut cultivars rapidly increases. This increase in hazelnut acreage is accompanied by renewed interest in developing control strategies for bacterial blight. Information on susceptibility of hazelnut cultivars to Xac is limited, partially due to lack of verified methods to quantify hazelnut cultivar response to artificial inoculation. In this research, Xac inoculation protocols were adapted to two hazelnut growing environments to evaluate cultivar susceptibility: *in vitro* tissue culture under sterile and controlled conditions, and *in vivo* potted tree conditions. Five hazelnut cultivars were evaluated using the *in vitro* inoculation protocol and seven hazelnut cultivars were evaluated using the *in vivo* inoculation protocol. Under *in vitro* conditions, there were severe bacterial blight symptoms on each cultivar consistent with those seen in the field, but no significant differences in the susceptibility of the newly released cultivars were observed compared to known Xac-susceptible cultivar (“Barcelona”). Under *in vivo* conditions, the proportion of necrotic buds were significantly higher in “Jefferson” and “Dorris” compared to all of the other tested cultivars, including “Barcelona.” The symptom progression seen *in vivo* mirrored the timing and symptom progression of bacterial blight reported from field observations. The *in vitro* conditions significantly reduced the amount of time required to measure the inoculation efficiency compared to the *in vivo* environment and allowed for greater replication. Further studies on the effects of Xac can use the results of these experiments to establish a dose–response model for bacterial blight, a wider range of germplasm can be tested under *in vitro* conditions, and management strategies that can be evaluated on large populations of new cultivars using the *in vivo* methods.

## Introduction

Bacterial blight of hazelnut caused by *Xanthomonas arboricola* pv. *corylina* (Xac) (Miller et al., 1940; Vauterin et al., 1995) is one of the most economically impactful diseases in commercial production of European hazelnut (*Corylus avellana* L.) worldwide. Bacterial blight is the second most important disease in the Oregon hazelnut industry behind the devastating fungal disease eastern filbert blight (EFB), which was inadvertently introduced from its native range in the eastern U.S. (Johnson et al., 1996). The hazelnut production acreage in Oregon has more than doubled since the release of EFB-resistant cultivars from 2007 to 2021 with roughly 34,000 Ha currently under cultivation (Pacific Agricultural Survey LLC, 2021). The rapid increase in planting enabled by EFB-resistant cultivars has come with many biotic and abiotic challenges, including increased reports of bacterial blight in young orchards.

*Xanthomonas arboricola* pv. *corylina* is a highly host-specific pathogen that exclusively causes bacterial blight in hazelnuts (*Corylus* spp.) (Miller et al., 1940). Bacterial blight symptoms are found on leaves, buds, twigs, trunks, and occasionally nuts, primarily on young hazelnut trees between 1 and 4 years old (Miller et al., 1949; Scortichini et al., 2002; Lamichhane and Varvaro, 2014; Kałuzna et al., 2021). It has been shown that Xac may reside epiphytically on asymptomatic plant tissues including under bud scales for extended periods without inducing symptoms (Pisetta et al., 2016). Suboptimal soil in planting sites, dryland production, and excess nitrogen in the soil have been associated with bacterial blight infection on hazelnuts (Moore, 1974; Lamichhane et al., 2013; Olsen, 2013; Pisetta et al., 2016). The disease can be difficult to detect in young hazelnut trees until the symptoms have had a detrimental effect in orchards, making this a challenging disease to manage and study in the field.

Planting resistant cultivars has been suggested as the best control method for managing bacterial blight since the disease was first described, especially in conjunction with sanitary cultural practices and timely copper sprays (Barss, 1913; Miller et al., 1949; Prunier et al., 1976; Lamichhane and Varvaro, 2014). However, observations on cultivar susceptibility reported in literature pertain to “legacy” varieties that are rarely planted in Oregon because they are highly susceptible to EFB (Olsen et al., 2013). The expansion of the hazelnut industry using cultivars with genetic resistance to EFB (McCluskey et al., 2011; Mehlenbacher et al., 2011, 2014, 2016, 2018, 2019) has exposed knowledge gaps in how to best manage the disease. No data exists on the susceptibility of these new EFB-resistant hazelnut cultivars to bacterial blight (Pscheidt and Ocamb, 2021).

Studies have been carried out on the Xac pathogen describing phenotypic, biochemical, and molecular qualities of the bacterium and pathogenicity testing of the pathogen on hazelnuts (Scortichini et al., 2002; Puławska et al., 2010; Prokić et al., 2012; Lamichhane and Varvaro, 2014; Webber et al., 2020). Bacterial blight infection requires pathogen presence and ideal environmental conditions and timing during specific growth stages of the hazelnut tissue to permit infection (Miller et al., 1949; Moore, 1969). The primary infection period of bacterial blight in Oregon orchards is in the late fall and early winter months during rainy and wet conditions (Miller et al., 1949). During some growing seasons, the disease is highly problematic in young orchards. After the fall infection period, symptoms of bacterial blight first appear the following spring from early to mid-April through early June in Oregon (Miller, 1937; Moore, 1974). Infections in spring may continue to develop and damage trees through the current growing season or even into subsequent seasons, but there are no new infection periods during the summer months (Miller et al., 1949; Scortichini et al., 2002; Prokić et al., 2012; Lamichhane and Varvaro, 2014).

Evaluating the susceptibility of different cultivars to the Xac pathogen under controlled field inoculations is a challenging prospect with environmental variability playing an important role along with the prolonged disease cycle. Scortichini et al. (2002) evaluated response to bacterial blight in three Italian hazelnut cultivars in the field inoculated with 31 Xac isolates. Buds entering dormancy were injected with each isolate and disease symptoms were assessed months later in spring. There was no difference found in susceptibility among the three hazelnut cultivars tested. Greenhouse experiments with potted trees also have been used under the conditions needed for successful inoculation in small-scale experiments (Miller et al., 1949; Prokić et al., 2012), but the capacity for large-scale experiments to achieve high replication of treatments is challenging.

As an alternative to inoculation experiments on potted or planted trees, micropropagation can been used to more rapidly produce many plant replicates in a controlled environment for disease screening (Barlass et al., 1986; Duron, 1987; Brisset et al., 1988; Scheck et al., 1997; Tripathi et al., 2008; Chandra, 2010). Tissue culture has had a great impact on the ability to produce large quantities of true-to-type, disease-free plantlets in a relatively short period of time with year-round application. Hazelnuts were first propagated in an *in vitro* system in 1975 (Radojevic et al., 1975), and many improvements have been made to optimize propagation since. Yu and Reed (1993) tested a variety of basal media and carbon sources and found that Driver–Kuniyuki Walnut (DKW) medium produced optimal shoot multiplication for hazelnuts. The DKW micropropagation media was adjusted and improved over the years, and the most recent *Corylus* media was formulated in 2016 for optimal hazelnut growth with DKW as the basis (Nas and Read, 2004; Bacchetta et al., 2008; Akin et al., 2017).

In this study, we tested susceptibility of new hazelnut cultivars with *in vivo* and *in vitro* inoculation experiments. For the *in vitro* susceptibility study, five hazelnut cultivars were micropropagated and used in inoculation experiments with Xac. These cultivars represented four of the new releases from the Oregon State University breeding program with single gene resistance to EFB including “Jefferson” (Mehlenbacher et al., 2011), “Dorris” (Mehlenbacher et al., 2013), “McDonald” (Mehlenbacher et al., 2016), and “Wepster” (Mehlenbacher et al., 2014), and also one known susceptible legacy cultivar “Barcelona” (Olsen et al., 2013). Tissue culture was used in a controlled environment to investigate the potential for developing a rapid screening technique for disease on hazelnut explants. For the *in vivo* susceptibility testing, potted trees were maintained outdoors and inoculated using the bud injection method reported by Scortichini et al. (2002) on the same cultivars used for the *in vitro* study with two additional cultivars, “Yamhill” and the pollinizer “York” (Mehlenbacher et al., 2009, 2018). This was the first potted tree inoculation experiment to examine response to bacterial blight in the new Oregon State University hazelnut cultivars. The data from each inoculation system were analyzed to quantify the incidence of disease and symptoms in each of the hazelnut cultivars.

## Materials and Methods

### Plant Material

The hazelnut cultivars evaluated *in vitro* included “Barcelona” (bacterial blight susceptible control), “Jefferson,” “McDonald,” “Wepster,” and “Dorris.” The tissue culture plant materials were maintained in the Oregon State University Horticulture Department Tissue Culture Lab in Corvallis, OR, USA. The explants were propagated for the experiment using the 2016 *Corylus* micropropagation medium for 6 weeks [NH_4_NO_3_, MgSO_4_·7H_2_O, K_2_SO_4_, KH_2_PO_4_, CaCl_2_·2H_2_O, Ca(NO_3_)_2_·4H_2_O, DKW-micronutrients, DKW vitamins, 2 mg L^−1^ Murashige and Skoog Thiamine, 200 mg L^−1^ Sequestrene-Fe 138, 6-benzylaminopurine (5 mg L^−1^), adjusted to pH 5.2, and solidified with agar (6 g L^−1^) (PhytoTechnology Laboratories A1111, Lenexa, KS, USA)] (Akin et al., 2017). The explants from the 5 cultivars were then transferred to culture tubes containing 10 ml of water-agar medium (sterile DI water solidified with agar 6 g L^−1^) (PhytoTechnology Laboratories A1111, Lenexa, KS, USA). Water-agar medium was used to maintain plants after inoculation because Xac grew on micropropagation media in preliminary experiments.

In the potted tree *in vivo* evaluation, “Yamhill” and “York” were included along with the five cultivars tested *in vitro*. The 6 EFB-resistant cultivars were grown as micropropagated trees purchased as plugs (North American Plants, LLC, McMinnville, OR, USA) in 2017 and 2018 raised in pots (2.6 L) using Metro Mix 840 PC potting medium (Sun Gro Horticulture Ltd., Agawam, MA, USA), under greenhouse conditions (16:8 L:D, 25°C). One group of trees was 2 years old at the time of inoculation. They were potted and maintained in a greenhouse during the spring of 2017 and then were held outdoors until inoculation. Potted trees were regularly irrigated and were provided with 20 g slow-release fertilizer every 6 months (15-9-12, Osmocote^®^ Plus, Maryville, OH, USA). A second group of trees were 1 year old at the time of inoculation. They were potted in the spring of 2018, given slow-release fertilizer, and maintained in the greenhouse. These trees were acclimated to the outdoors and went into dormancy in the fall of 2018. The legacy cultivar “Barcelona” (known susceptible) was not available from tissue culture, so stool-bed layered trees were potted up and cared for in the same manner as the other trees.

### Inoculum Preparation

*Xanthomonas arboricola* pv. *corylina* strain JL2600 was isolated from a commercial hazelnut orchard in the Willamette Valley, OR, USA, characterized, and shown to be virulent (Webber et al., 2020). The strain was formulated as a lyophilized powder for inoculation of hazelnut. JL2600 was cultured for 5 days at 27°C on several plates of glucose, yeast, calcium carbonate agar (GYCA) (Prokić et al., 2012). Bacterial lawns were recovered from the media with a spatula, mixed with powdered skim milk [38% (w/v)], and frozen at −80°C prior to lyophilization using a FreeZone 6 system Freeze Drier (Labconco Co. Kansas City, MO, USA). The freeze-dried product was ground to a fine powder and stored at −80°C (Johnson et al., 1993; Rothleutner et al., 2014). The titer of the freeze-dried formulation of JL2600 was verified routinely and was consistent among all experiments.

### *In vitro* Inoculation Procedure

Twenty replicate explants per cultivar were treated with JL2600 or sterile DI water as a negative control. The *in vitro* inoculation experiment was repeated three times. Explants were removed from culture tubes and the apical meristem was removed aseptically with a scalpel to expose the vascular tissue. The explants were swirled for 10 s in a suspension of JL2600, at a concentration of 1 × 10^7^ colony forming units (CFU)/ml, or sterile deionized (DI) water, and the excess liquid was allowed to drip off. The lyophilized bacterium was suspended in sterile DI water and incubated for 1 h at room temperature prior to inoculation. After treatment, explants were placed in water agar in culture tubes and maintained in a growth chamber at 25°C with a 14:10 L:D photoperiod for the duration of the 9-week experiment.

### *In vitro* Symptom Assessment

All changes in the appearance of the treated explants were observed and recorded to develop a screening method under *in vitro* conditions. Symptoms were evaluated once a week for the duration of the experiment during each of the three replications. The cultivar evaluation experiment was terminated at 8 weeks post-inoculation (wpi). The pathogen was isolated from symptomatic tissues and verified to fulfill the postulate of Koch. Lesions were rated as any imperfections or blemishes present on the leaf surface. During the initial evaluation on the date of inoculation (0 wpi), minor blemishes that were naturally present on the leaves of tissue culture plantlets were counted. As the weeks progressed, leaves with lesions, chlorosis, or chlorotic patches were counted and recorded on each explant. Necrotic leaves or leaves with developing necrotic patches were also counted on each explant. The symptoms on each leaf were assigned to a category based on which symptom was dominant (lesions, chlorotic, or necrotic). At each time point, asymptomatic leaves also were counted to allow calculation of the proportion of symptomatic leaves.

### *In vitro* Potted Tree Inoculation

Inoculations were carried out by administering the treatments with a needle syringe to individual buds on each tree using the method of Scortichini et al. (2002). Briefly, the cultivars were divided into JL2600 and control treatment groups with an equal number of trees in each. A suspension of JL2600 at 1 × 10^8^ CFU/ml was prepared in a sterile 10 mM phosphate buffer, pH 7.0. The negative control treatment was sterile phosphate buffer.

The trees were labeled with their respective treatments. Between 7 and 20 buds were marked on each tree, depending on the number of buds available, with a twist tie marker placed at the bottom of the branch with treated buds. The buds selected for treatment were injected with 10 μl of sterile phosphate buffer or JL2600 (1 × 10^8^ CFU/ml) under the bud scales until runoff using a sterile 1-cc hypodermic syringe fitted with a 28-G needle (Webber et al., 2020).

The total population of treated cultivars consisted of 880 trees. There were 240 two-year-old trees, 600 one-year-old trees, and 40 layered “Barcelona” 1-year-old trees. The treatments were carried out during the first week of November 2018 over 4 consecutive days. Each day, an equal number of Xac-inoculations and sterile phosphate buffer control treatments were administered for each cultivar and age. New inoculum and phosphate buffer controls were used each day, and the concentration of bacteria was consistent. The treatments were kept separate during inoculation to avoid cross contamination while the inoculum was still wet. One week after inoculation, the JL2600-treated trees and the negative control trees of each cultivar were arranged into a completely randomized design. After inoculation, the trees were held over winter on an outdoor pad at North Willamette Research and Extension Center, Oregon State University, Aurora, OR, USA. Trees were monitored periodically throughout the dormant season and checked weekly as spring approached and the buds began to swell and break.

### *In vivo* Potted Tree Symptom Assessment

Evaluation of symptoms was performed in the first week of May 2019. Inoculated buds were rated as infected by the presence of lesions and necrotic tissue on the buds, petioles, and emerging leaves of each tree in a similar manner to the *in vitro* study. A random sample of symptomatic and asymptomatic tissue from each cultivar and treatment was collected to re-isolate inoculated bacteria to fulfill the postulate of Koch. The re-isolated bacteria were identified using dilution plating on the semi-selective growing medium GYCA and sequence analysis of the housekeeping gene *gyrB* to detect unique polymorphisms in JL2600 (Webber et al., 2020).

### Statistical Analyses

The open-source statistical environment R (R Core Team, 2020) was used for all statistical analyses and to produce the associated figures. Packages utilized include ‘dplyr’ for data manipulation (Wickham et al., 2020) and ‘ggplot2’ for graphics (Wickham, 2009). For the *in vitro* study, the proportion of symptomatic leaves was analyzed with a two-column matrix containing the proportion of symptomatic and healthy leaves as the response variable in a binomial generalized linear repeated measures model (GLM). The GLM used a binomial error distribution and interactive predictor variables between cultivar, treatment, and week after a generalized linear mixed effects model (GLMM) using glmer from the package ‘lme4’ (Bates et al., 2015) with a random effect on subject (plant) showed negligible (near zero) random effect variance, thus justifying the simpler GLM. An analysis of deviance chi-squared Wald test was performed on the GLM, followed by Tukey's multiple comparisons test from the package ‘emmeans’(Lenth, 2021) to evaluate separation of means for the significant GLMM. Results were analyzed for the *in vivo* potted tree inoculation using GLMM with glmer in the package ‘lme4’(Bates et al., 2015). The response variable for the GLMM was the proportion of necrotic buds represented by a two-column matrix with the number of buds infected (symptomatic) and the number of healthy buds of the total inoculated. The fixed effect predictors for the model were the interaction between treatment and cultivar, and age of tree at the time of inoculation was included as a random effect. The error distribution family selected for the GLMM was binomial. Analysis of deviance and Tukey *post-hoc* comparisons were performed on the GLMM for the *in vivo* data as described above for the GLM for the *in vitro* data.

## Results

### *In vitro* Inoculations

**Bacterial blight symptoms under the controlled conditions were consistent with symptoms observed in the** ***in vivo* inoculation, and no differences in cultivar susceptibility were detected**.

**Figure 1:**
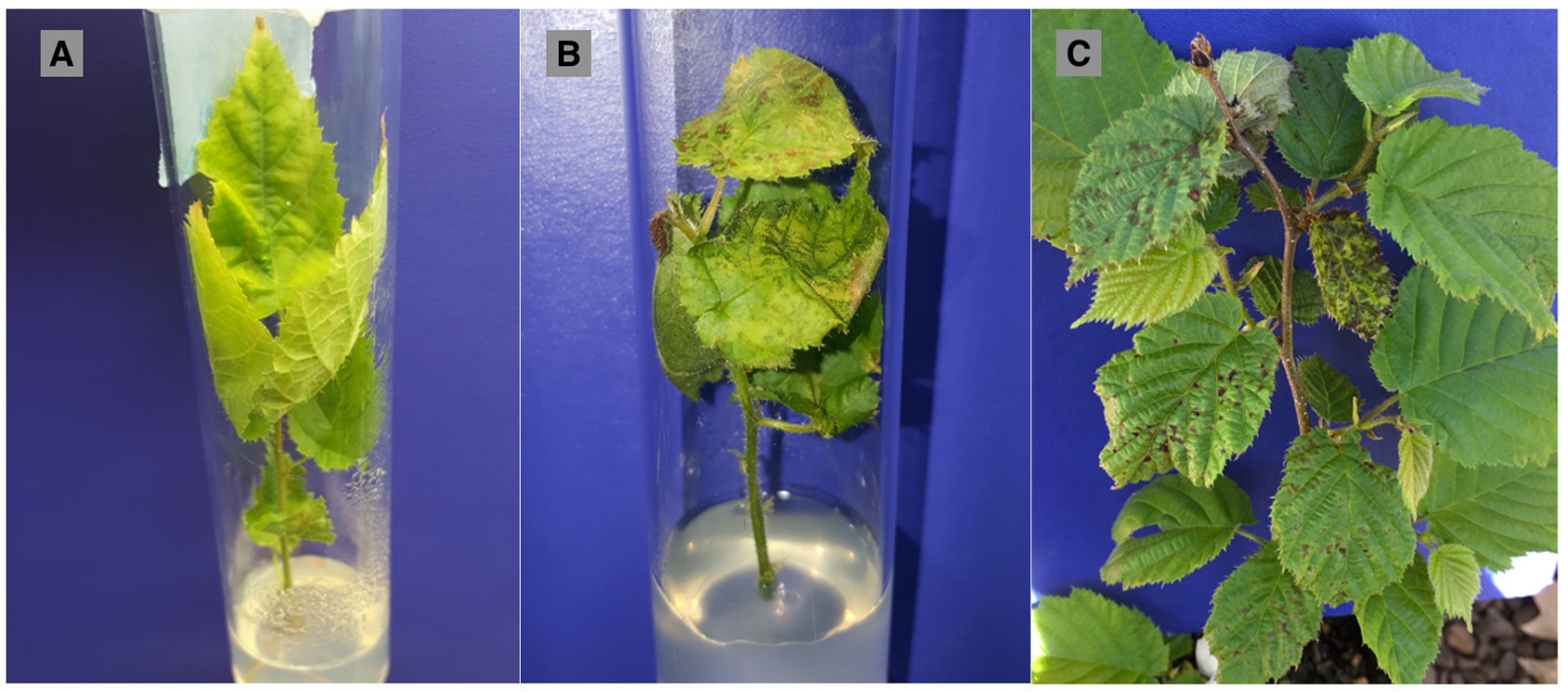
Symptoms following controlled inoculations of both *in vitro* and *in vivo* plant material. **(A)** “Barcelona” explant from the *in vitro* experiment treated with sterilized deionized water (control) showing chlorosis from growing on the water-agar medium, **(B)** “Jefferson” explant from *in vitro* experiment showing severe chlorosis and water-soaked lesions at 5 weeks post-inoculation with *Xanthomonas arboricola* pv. *corylina* strain JL2600, and **(C)** “Jefferson” potted tree (*in vivo*) showing severe chlorosis and water-soaked lesions at 6 months post-inoculation with JL2600.

**Figure 2:**
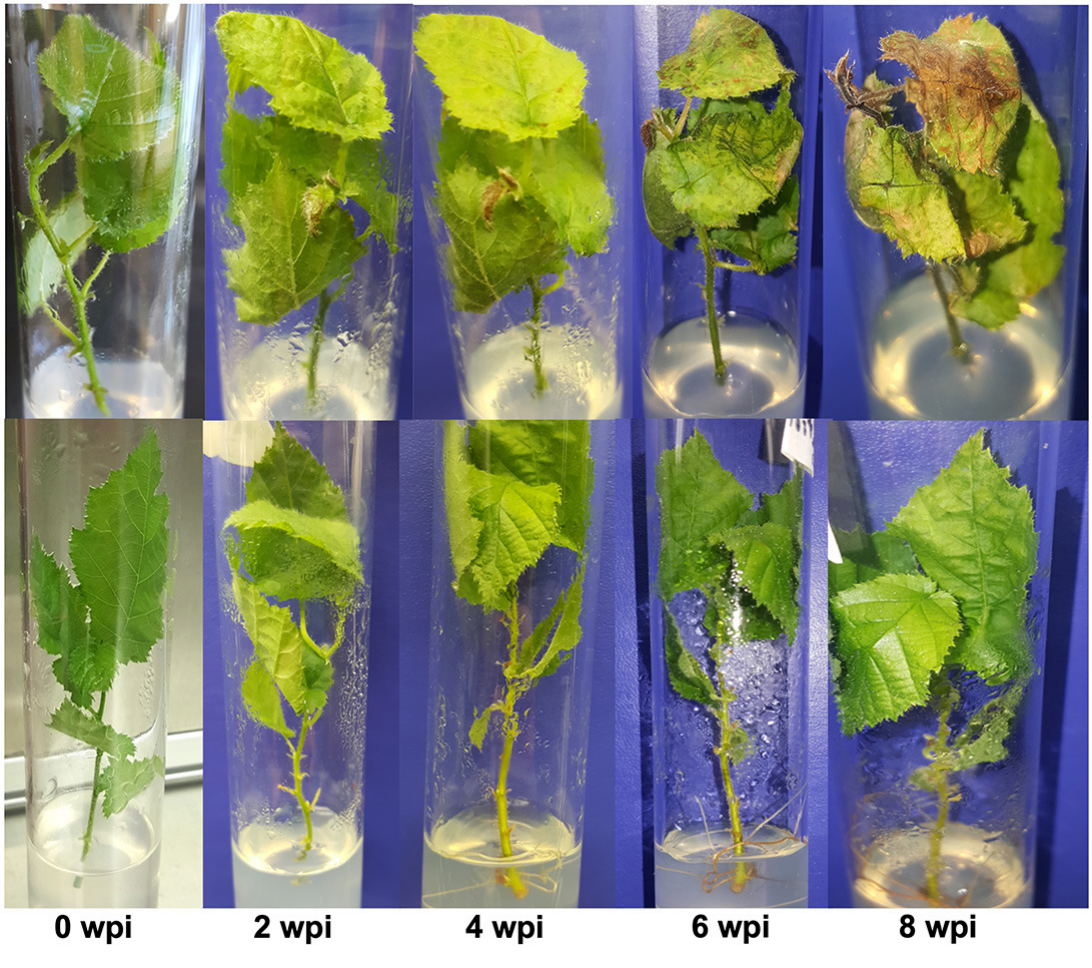
Symptom progression on known bacterial blight susceptible legacy cultivar “Barcelona” following *in vitro* inoculations with Xac-JL2600 (top row) and water control treatment (bottom row). The Xac-JL2600 treatment (top row) shows a gradual progression of disease symptoms starting at 0 wpi (weeks post-inoculation) with the explant free of visible blemishes or symptoms, slight chlorosis and water-soaked lesion formation beginning at 2 wpi and becoming more prominent by 4 wpi. By 6 wpi, the chlorosis and water-soaked lesions were severe and turning necrotic, moving into the stem by 8 wpi. The water control treatment (bottom row) showed relatively few changes in leaf symptoms throughout the evaluation.

The response variable for the *in vitro* study was symptomatic leaves representing leaves that had lesions, chlorosis or necrosis. It was not possible during the weekly assessments of explants to confidently visually differentiate the physiological effects of progressing nutrient deficiency in explants growing in water-agar medium (Figure 1A), and symptoms due to progression of the disease (Figure 1B). Symptoms in the water controls were not related to infection or contamination by Xac, as no bacteria were ever isolated from the water control explants at the conclusion of the *in vitro* experiment. Thus, “symptomatic” hereafter for the *in vitro* study refers to symptoms of bacterial blight in the JL2600-treated explants, and to bacterial blight-like symptoms in explants treated with water only. Symptom progression in known susceptible “Barcelona” for the JL2600 treatment and the water control treatment throughout the 8-week evaluation period for the *in vitro* study is shown in Figure 2.

**Figure 3:**
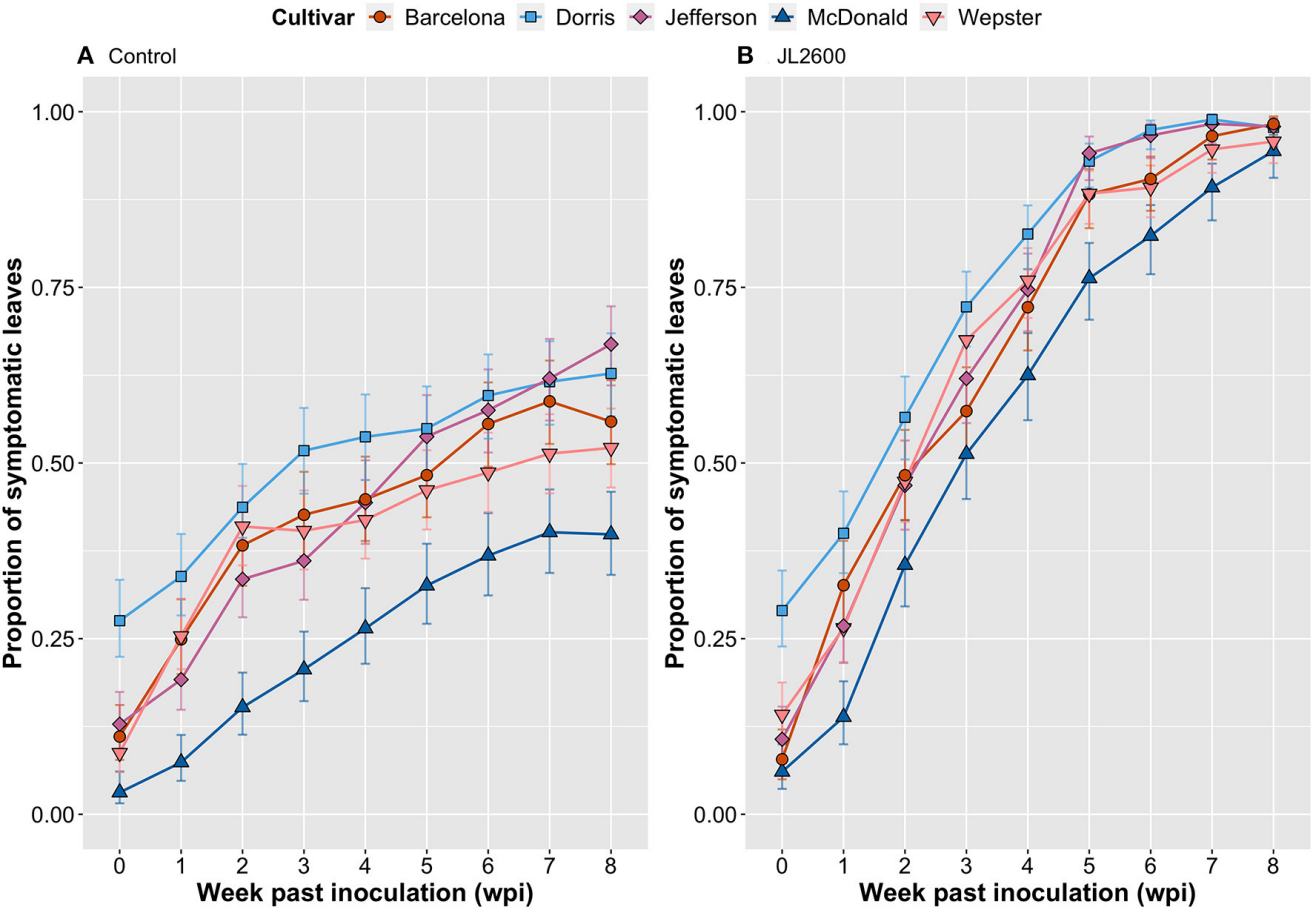
Comparison of host responses over an 8-week evaluation period of five cultivars of hazelnut, propagated as tissue cultured explants on water-agar medium, and dip inoculated with sterile-deionized water [**(A)**. Control], or the hazelnut bacterial blight pathogen *Xanthomonas arboricola* pv. *corylina* strain JL2600 [**(B)**. JL2600]. Data points represent the proportion of total leaves on an explant that were symptomatic. Vertical bars represent 95% CI.

The GLM indicated that there were significant differences in the proportion of symptomatic leaves on explants depending on cultivar alone (cultivar; Table 1). This may be explained by the observation that the cultivars may have had different tolerances to the water-agar medium. For example, at the time of treatment (0 wpi), ‘Dorris’already had a significantly higher proportion of symptomatic leaves compared to other cultivars in both the JL2600-treated and the water-treated explants [Tukey's honestly significant difference (HSD); *p* < 0.05; Figure 3]. The predictor treatment had a significant effect on the proportion of symptomatic leaves (treatment; Table 1), and it was clear that even though bacterial blight-like symptoms were present in the bacteria-free water controls and increased over time, that JL2600-inoculated explants showed a more consistent rate of increase of disease symptoms on leaves (Figure 3B).

**Table 1:** Analysis of deviance results for the generalized linear model for the proportion of symptomatic leaves of explants in the *in vitro* study.

**Predictor**	**d.f**.	***χ^2^***	***p*** **>** ***χ^2^***
Cultivar	4	552.153	<2.2e-16
Treatment	1	1254.032	<2.2e-16
Wpi	8	2754.182	<2.2e-16
Cultivar:treatment	4	7.786	0.099
Cultivar:wpi	32	95.493	3.089e-11
Treatment:wpi	8	621.395	<2.2e-16
Cultivar:treatment:wpi	32	52.199	0.014

For the GLM interaction terms, inoculation treatment with water or JL2600 had a similar effect on all cultivars (cultivar:treatment; Table 1), suggesting that there was not a clear difference in susceptibility to Xac among cultivars under the tested experimental conditions. However, cultivars had different proportions of symptomatic leaves depending on the wpi (cultivar:wpi; Table 1), but again, this result reflects on the different reaction of cultivars to the water-agar medium over time. The treatment effect on symptoms also depended on the week in which leaves were evaluated (treatment:wpi; Table 1), and finally, depending on the week, treatments had significantly different effects on the different cultivars (cultivar:treatment:wpi; Table 1). There was a time delay before this latter interaction effect began to appear. There were no significant differences between the proportion of symptomatic leaves between the JL2600-treated explants and their respective control explants of any cultivar at 0 wpi or 1 wpi (Tukey's HSD; *p* > 0.05; Figure 3). The first significant treatment effect where JL2600-treated cultivars had a higher proportion of symptomatic leaves compared to water-treated control was at 2 wpi with “McDonald” and “Dorris” (Tukey's HSD; *p* < 0.05; Figure 3). At 2 wpi, characteristic bacterial ooze was observed on site where the meristem had been removed in each of the cultivars. After 3 wpi, all JL2600-treated cultivars had a significantly greater proportion of necrotic leaves than their respective controls (Tukey's HSD; *p* < 0.05; Figure 3). There were significant differences between the proportion of symptomatic leaves for some of the Xac-treated cultivars at different time points or wpi, but the disease progressed at a very similar rate in all Xac-treated cultivars (Figure 3B). All cultivars treated with water (controls) had an increasing proportion of leaves showing bacterial blight-like symptoms of necrosis from the time of treatment to 3–4 wpi, and subsequently the rate of increase in the proportion of symptomatic leaves on water-treated explants slowed (Figure 3A). At 8 wpi and the end of the trial, the water-treated controls had a significantly lower incidence of symptomatic leaves compared to JL2600-treated explants, which tended to be completely necrotic by this time (Tukey's HSD; *p* < 0.05; Figure 3).

Strain JL2600 was consistently isolated from inoculated explants at population densities ranging from 1 × 10^4^ to 1 × 10^7^ CFU/explant. Despite the increase in bacterial blight-like symptoms observed on the water-treated control explants over time for each cultivar, no bacteria were re-isolated on GYCA from these explants. Thus, symptoms on control explants were interpreted to indicate stress in response to growing in water-agar medium devoid of nutrients.

### *In vivo* Inoculation

Evaluation shows “Dorris” and “Jefferson” having the highest disease incidence compared to “Barcelona,” “Yamhill,” “McDonald,” “Wepster,” and “York.”

**Figure 4:**
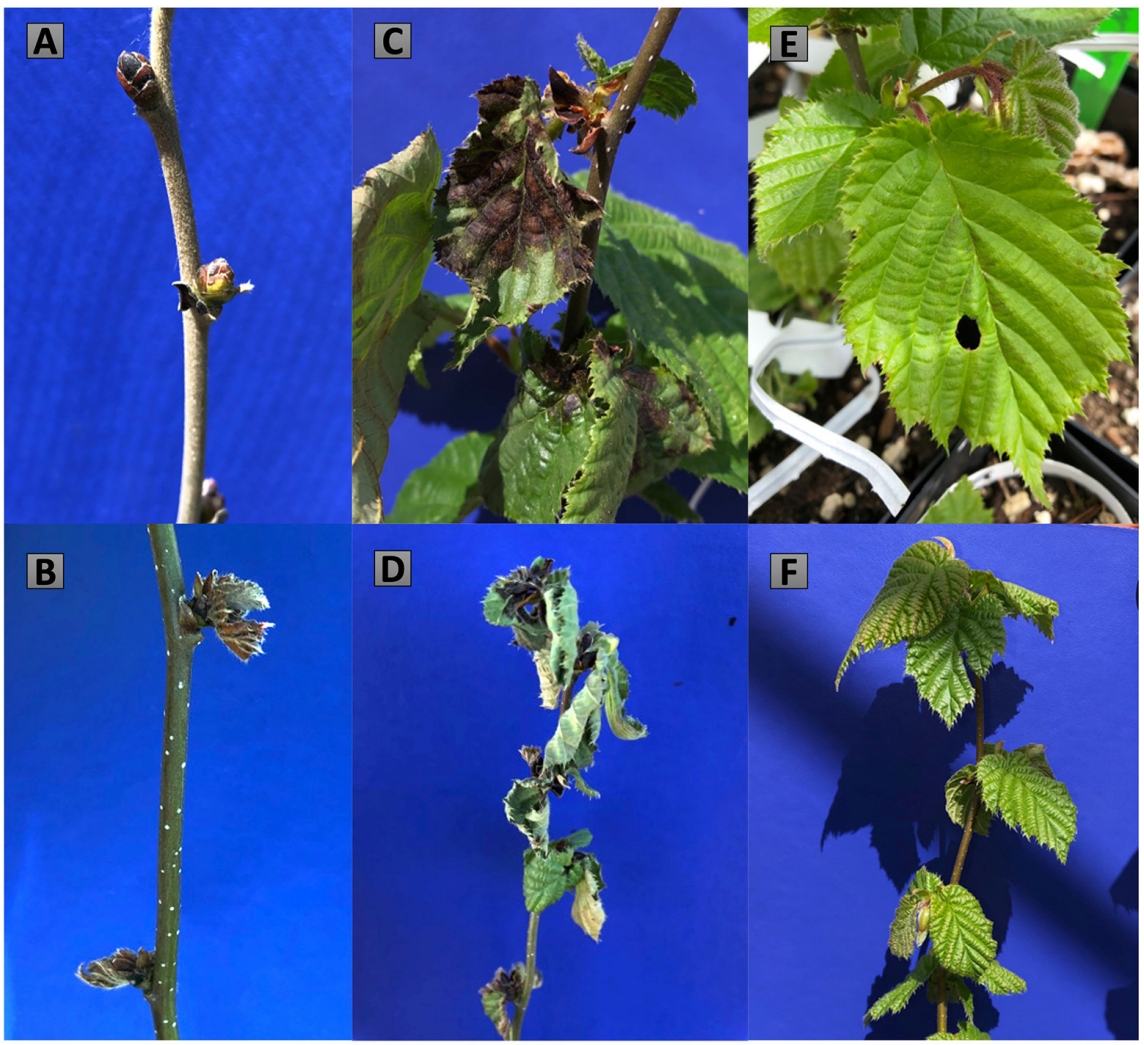
Disease symptoms 6 months after the *in vivo* inoculations with Xac-JL2600 treatment **(A–D)** and the sterile phosphate buffer control treatment **(E,F)**. Xac-JL2600 symptoms presented as **(A)** necrotic buds, **(B)** partially opened necrotic buds, **(C)** water-soaked lesions and necrotic spots on newly emerged shoots, and **(D)** the dieback of newly emerged shoots. The water control treatment showed **(E)** visible holes where the inoculation needle punctured the leaf primordia and **(F)** emerging shoots developing free of symptoms.

The first symptoms of bacterial blight in the *in vivo* trial were observed on April 16, 2019, 160 days post-inoculation, when the trees were in the half-inch green leaf development stage. Symptoms appeared as failure of buds to open, death of partially opened buds, and water-soaked lesions on emerging leaves (Figures 1C, 4). Symptoms progressed for another 3 weeks and were evaluated on May 6, 2019. Buds treated with water did not show any symptoms of bacterial blight, but in some cases the leaves that developed from buds inoculated with the sterile phosphate buffer (controls) had holes in them where the syringe had pierced the leaf primordia in the bud.

**Figure 5:**
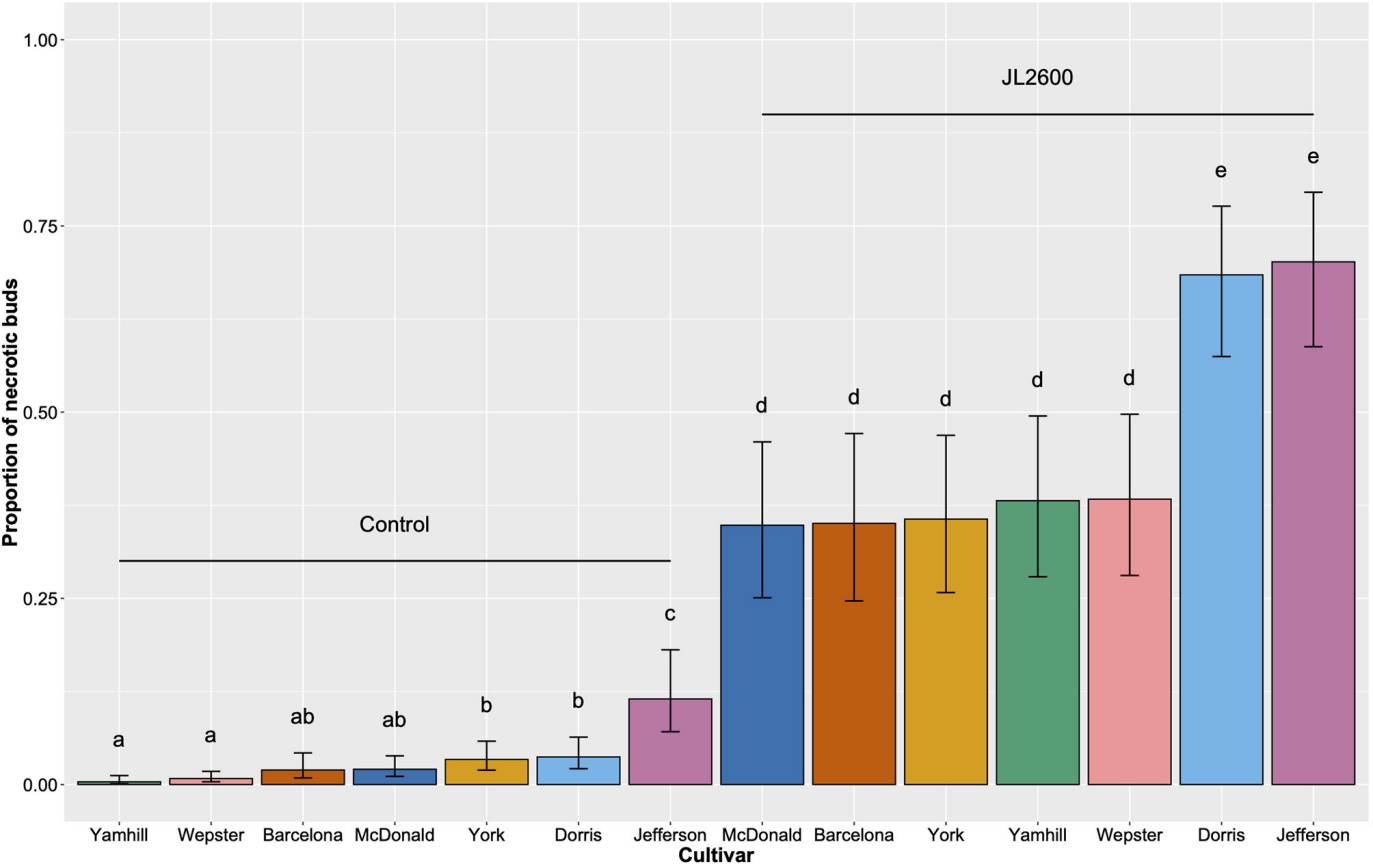
Comparison of the proportion of necrotic buds across seven different cultivars of hazelnut consisting of 880 potted trees (*in vivo*) maintained outdoors. Each tree had between 7 and 20 buds inoculated by injection in fall 2018 with sterile phosphate buffer (Control), or the bacterial blight pathogen *Xanthomonas arboricola* pv. *corylina* strain JL2600. The population was evaluated in spring 2019. Vertical bars represent 95% CI. Different letters above bars indicate significant differences by Tukey's HSD (*p* < 0.05).

All of the predictors from the GLMM had a highly significant influence on the model demonstrating that cultivar, treatment, and the interaction between cultivar and treatment had significant effects on the proportion of necrotic buds on the potted trees (Table 2). In each cultivar, the incidence of necrotic buds among all of the buds inoculated with JL2600 was significantly different than the proportion of necrotic buds on the sterile phosphate buffer controls (Tukey's HSD; *p* < 0.05; Figure 5). The known susceptible “Barcelona” trees had a mean incidence of bud necrosis of 0.350 (±0.058 SEM) on buds inoculated with JL2600, whereas the incidence of bud necrosis on control buds was 0.019 (±0.007 SEM) (Figure 5). Mean incidences of bud necrosis of inoculated “McDonald” (0.348 ± 0.054 SEM), “Wepster” (0.383, ±0.052 SEM), “Yamhill” (0.381 ± 0.056 SEM), and “York” (0.356 ± 0.055 SEM) showed no difference in disease incidence compared to “Barcelona” and each other (*p* > 0.05; Figure 5). However, the mean incidence of necrotic buds for JL2600-inoculated “Jefferson” (0.702 ± 0.053 SEM) and “Dorris” (0.684 ± 0.052 SEM) had a significantly greater disease incidence than “Barcelona” and all other JL2600-inoculated and control trees (Tukey's HSD; *p* < 0.05; Figure 5). No difference in the incidence of necrotic buds was found with JL2600-inoculated “Jefferson” and “Dorris” (Tukey's HSD; *p* > 0.05; Figure 5). Within the control treatment, “Jefferson” had the highest mean proportion of necrotic buds (0.115 ± 0.028), which was significantly greater than each of the other control cultivars (Tukey's HSD; *p* < 0.05; Figure 5). “Yamhill” and “Wepster” controls had the lowest proportion of necrotic buds of any cultivar/treatment combination (0.004 ± 0.002, and 0.008 ± 0.003, respectively; Tukey's HSD; *p* < 0.05).

**Table 2:** Analysis of deviance results for the generalized linear mixed model for the proportion of symptomatic buds on potted trees in the *in vivo* study.

**Predictor**	**d.f**.	***χ^2^***	***p*** **>** ***χ^2^***
Cultivar	6	419.969	<2.2e-16
Treatment	1	1309.452	<2.2e-16
Cultivar:treatment	6	44.491	5.908e-08

Bacteria were recovered from symptomatic buds and tissue from the JL2600-treated trees at concentrations consistently 1,000-fold greater (1 × 10^9^ CFU/bud) than the concentration injected into the buds at inoculation (1 × 10^6^ CFU/buds). A lower concentration of bacteria (1 × 10^4^ CFU/bud) with the morphology of Xac were recovered from a random sample of asymptomatic control buds. These occurrences of Xac on the asymptomatic control buds suggest that bacteria from the JL2600-treated trees splashed onto control trees post-inoculation after the potted trees were arranged into a complete randomized design and were left over winter. These bacteria residing epiphytically on the asymptomatic bud surface is consistent with how Xac is naturally spread in an orchard environment. Amplification and Sanger sequencing of *gyrB* in the recovered bacteria from each treatment showed single nucleotide polymorphisms that were consistent with strain JL2600.

## Discussion

Hazelnut cultivars propagated and inoculated under *in vitro* conditions were useful for examining pathogenicity of Xac and could form the basis for a protocol for rapid disease screening for hazelnut cultivars. After inoculation under *in vivo* conditions, each of the EFB-resistant cultivars was found to be susceptible to bacterial blight infection with disease incidence equal to or greater than the known susceptible cultivar Barcelona. The *in vitro* evaluation also supported these results. While the two inoculation methods were used in this study were using different forms of plant material and different inoculation methods, each evaluation system consistently demonstrated bacterial blight susceptibility and disease symptoms.

The cultivar “Barcelona” was classified under natural conditions as moderately to highly susceptible to bacterial blight and was included in this study as a known susceptible (Barss, 1927; Miller et al., 1949; Pscheidt and Ocamb, 2021). Previous bacterial blight investigations reported the cultivar response to bacterial blight based on observations under natural conditions of infection, but with no formal quantification of disease. Several hazelnut cultivars have been reported under field conditions as having a degree of bacterial blight resistance such as pollinizers “Daviana” and “Hall's Giant”; however, with poor nut quality and EFB susceptibility, they are no longer widely planted (Miller et al., 1949; Prunier et al., 1976; Thompson et al., 1996). It would have been advantageous to include these purportedly resistant cultivars in this study, but plant material of these legacy pollinizer cultivars was unavailable. However, under each inoculation system evaluated in this study, the disease incidence for “Barcelona” supports the observation of at least moderate susceptibility. “Yamhill,” “McDonald,” “Wepster,” and “York” showed no difference in disease incidence to “Barcelona” suggesting that they too are moderately susceptible to bacterial blight. “Dorris” and “Jefferson” had the highest bacterial blight disease incidence, significantly greater than any other cultivar, suggesting they are highly susceptible to bacterial blight under the conditions evaluated. These recommendations of susceptibility are based on high replications of 1-year old and 2-year old *in vivo* individuals; however with only 1 year of data, it is worth noting the limitations of this experiment in drawing broad conclusions of susceptibility. Further testing across multiple growing seasons and environments are needed to make comprehensive recommendations.

The *in vivo* inoculation methods provided a quantitative measurement of buds infected out of total buds inoculated on each replicate, while ensuring that each bud was wounded and inoculated with the same concentration of the pathogen. However, this method was tedious and time consuming. Future potted tree or field inoculation experiments could evaluate spray-inoculation of Xac onto freshly pruned trees. Belisario et al. (1999) used this mass spraying inoculation method without wounding when evaluating a 1-year-old seedling population of *Juglans* species for disease resistance to walnut blight caused by *Xanthomonas arboricola* pv. *juglandis* (Xaj). The resulting disease susceptibility on the different species was assessed using the percentage of seedlings with cankers on the stems and branches. In a recent review, Kałuzna et al. (2021) highlighted the similarities between Xac and Xaj suggesting that inoculation, identification, and management techniques successful on Xaj could also be informative if applied on Xac. The time saved by using this spray inoculation technique would allow for greater population sizes and could be incorporated into mass bacterial blight screening on hazelnut. However, wounding of the tree could potentially lower the power to detect cultivar resistance to bacterial blight, as wounding or injury is not required nor necessarily associated with natural bacterial blight infections on buds, though bacterial blight is associated with pruning wounds on branches and trunks (Miller et al., 1949). Future studies seeking to determine susceptibility of different cultivars may also benefit from less aggressive inoculation procedures, such as application of droplets or sprayed inoculum on uninjured buds. As bacterial blight is associated with drought stress, it would also be interesting to examine effects of deficit irrigation on spray-inoculated trees.

The potted tree inoculation simulated bacteria overwintering in the bud scale with an added wound to localize the infection, but this method took much effort to prepare trees for inoculation, and then a lot of time is needed to see symptoms to develop in the spring following fall inoculation. While environmental conditions in the greenhouse stage were under control, placement of trees outdoors post-inoculation meant that trees were subject to natural environmental conditions, such as non-uniform wind, rain, and frost events, which could influence development of the disease and affect repeatability of experiments. These inconsistent environmental conditions caused inadvertent wind and rainwater splash of JL2600 bacteria from JL2600-treated trees onto water-treated buds. This was discovered when low concentrations of Xac were recovered from randomly collected asymptomatic water-treated buds. Perhaps the winter dormancy period could be simulated and shortened in a more controlled environment by utilization of cold storage facilities for storing inoculated plants to satisfy chill hour requirements, at which point they could be brought back to the greenhouse to break bud for subsequent evaluation of disease symptoms.

In contrast with the *in vivo* experiments, the *in vitro* method to evaluate disease susceptibility is quick, low cost, easily replicated, and allows greater control over environmental conditions compared to field and potted tree inoculations. Previous studies found tissue culture to be an effective method for high-volume screening of plant resistance to bacterial pathogens, and others found the results to be not comparable enough to field conditions to be an accurate screening tool (Brisset et al., 1988; Tripathi et al., 2008). Plant resistance and pathogenicity have been tested using tissue culture explants in systems such as: *Pseudomonas syringae* pv. *syringae* (*Pss*) on lilacs, *Xanthomonas campestris* pv. *musacearum* causing Xanthomonas wilt on bananas, and *Erwinia amylovora* causing fire blight on apples and pears (Duron, 1987; Brisset et al., 1988; Scheck et al., 1997; Tripathi et al., 2008). Scheck et al. (1997) screened the capacity of strains of *Pss*, isolated from several genera of host plants, to cause disease on lilac explants. In that assay, *Pss* did not grow on the MS tissue culture media so no special adjustments were needed for the culture media. In this study, Xac strains grew on the 2016 *Corylus* media (Akin et al., 2017). We maintained explants on water-agar medium, which may have amplified bacterial blight symptoms due to increased plant stress (Moore, 1974). The water-treated control explants showed stress symptoms indicative of nutrient deficiency, such as yellowing of the leaves, while maintained on water-agar medium. The stress symptom development in the controls leveled off over the duration of the experiment, but the appearance of these symptoms on the water-treated controls unrelated to Xac, reduced the contrast with disease symptoms in the Xac-treated explants.

*Pseudomonas syringae* pv. *syringae* produced uniform disease symptoms on the lilac explants maintained in MS tissue culture medium (Scheck et al., 1997). The disease symptoms on the lilac explants included water-soaked lesions on the leaves, vein, and petiole necrosis and tip dieback. The symptoms appeared as little as 2 days post-inoculation with a complete disease response after 14 days (Scheck et al., 1997). In this study, bacterial blight symptoms began to appear as early as 5 days post-inoculation with a complete disease response greater than the water controls at 3 wpi. The *in vitro* bacterial blight assay in hazelnuts takes twice as long as the *Pss* tissue culture assay, but it is a major improvement on previous methods of testing the disease response of Xac on hazelnut that can take up to 6 months. However, the *in vitro* test did not seem to give high resolution for discriminating susceptibility under our conditions as there was no clear difference seen among cultivars and the disease progressed at a very similar rate in all the tested cultivars (Scortichini et al., 2002; Prokić et al., 2012).

A rapid technique for evaluating banana cultivars to *Xanthomonas* wilt was developed using tissue culture methods compared to potted plants (Tripathi et al., 2008). There were eight cultivars of bananas tested and a gradient of susceptibility to *Xanthomonas* wilt were significant in both tissue culture and on potted plants (Tripathi et al., 2008). The cultivar disease incidence gradient of bacterial blight of hazelnut observed in the *in vivo* inoculation was not observed in the *in vitro* system. Differences in susceptibility might be seen under *in vitro* conditions at lower Xac inoculum concentrations while establishing dose-response curves for different cultivars. Additionally, evaluating cultivars *in vivo* or *in vitro* with several Xac strains with different levels of virulence could also introduce cultivar separation relevant to informing bacterial blight management decisions. As global hazelnut production increases and new cultivars of hazelnuts are developed, adapted methods of both *in vitro* and *in vivo* inoculation systems could be used to screen large populations of progeny or new cultivars for bacterial blight susceptibility. Such studies could provide a more global scope of cultivar susceptibility to Xac by including important European cultivars, and other cultivars that are being widely planted around the world.

## Data Availability Statement

The original contributions presented in the study are included in the article/supplementary material, further inquiries can be directed to the corresponding author.

## Author Contributions

NW secured the funding. SW provided tissue culture training, resources, and facilities. VS provided pathogen training, resources, and facilities. JW conducted the experiments and drafted the manuscript. All authors contributed to experimental design and writing and editing to the manuscript.

## Funding

This work was funded by a grant from the Oregon Hazelnut Commission and a donation from Jan and Linda Wepster to NW to support JW.

## Conflict of Interest

The authors declare that the research was conducted in the absence of any commercial or financial relationships that could be construed as a potential conflict of interest.

## Publisher's Note

All claims expressed in this article are solely those of the authors and do not necessarily represent those of their affiliated organizations, or those of the publisher, the editors and the reviewers. Any product that may be evaluated in this article, or claim that may be made by its manufacturer, is not guaranteed or endorsed by the publisher.
